# Golf and Physique

**Published:** 1908-08-08

**Authors:** 


### The Practitioner's Relaxations and Hobbies.

### GOLF AND PHYSIQUE.

A few years back some responsible persons interested in golf were curious and enterprising enough to circularise various prominent players noted for their long driving. The information sought was the secret of success in this particular department of the game—the answers received, and published, were singularly unanimous. All spoke, rather vaguely, of "knack," of acquired power, of very accurate timing of the stroke, of some quick flick of the wrists at an especially advantageous moment. Probably enough the value of these data was chiefly psychological. Any attentive newspaper reader must have remarked how common it is for men distinguished in any calling (our own is no exception) to get up at prize distributions and similar assemblies, and with a curious *naïveté*, or else mock modesty, to assure audiences that the way to attain eminence and expertness is simply by hard work and practice, omitting all reference to the obvious pre-requisites of exceptional intellectual ability and strength of character. Somewhat similarly, these athletes left out of count several natural causes of their power of driving a long ball, the most essential of which is perhaps physique.

Now Mr. Horace Hutchinson, who will be deservedly remembered by future generations of golfers almost as Nyren and Beckford are now remembered by cricketers and fox-hunters, is, no doubt, mainly responsible for this. He thinks strength, weight, and reach have not much to do with long driving, and has instanced the case of a consumptive near death who habitually out-drove a professional. Mr. Hutchinson it is who, in Uncle Remus's phrase, has "started up" the timing idea. When Vardon astonished everybody by his brassie shots, it was "very perfect timing" that was the probable explanation; the same when Massey became open champion. On this view, it is the velocity, and the velocity alone, of the club-head at the time of its impact on the ball which determines the length of the shot.

It may be so, but as happily golf is still a game, and not yet a dull mechanical craft, no one has yet measured the velocities club-heads commonly attain in the hands of different players, and so the theory lacks proof. On the other hand, the national taste for athletics causes in many people a keen desire to know the physical characteristics of noted players. We have the height, weight, and general build of such recorded in considerable detail. Now it may be said fairly confidently that no one will cavil at the selection of the following five as the prominent long drivers, in roughly chronological order, of the last twenty years. First, Douglas Rolland, who, often with borrowed clubs, could hit a gutta ball much further than nine out of ten men can now drive a rubber core; secondly, Mr. E. Blackwell, the reputed longest driver of the day; next, James Braid, seemingly able to drive two hundred and fifty yards whenever he likes; and fourth, Harry Vardon, whose power of making big carries has repeatedly enabled him to land his second shot over a bunker situated a quarter of a mile from the tee. Last, but not least, comes the late Mr. Tait, who, by his feat of driving more than three hundred yards, threw considerable doubt on his father's theory of a mathematical limit to the golfer's powers.

Now for their physique. A contemporary description of Rolland in his hey-day was couched in some such words as these: "Six feet high, twelve and a half stones in weight, and straight as a dart: a model for a sculptor." The writer goes on to speak of the exceptional strength of his wrists, attributable to his work as a stone mason. Mr. Blackwell is a giant, of course, six feet and some inches in height, and of huge frame. He is reported to use very heavy clubs. Braid, though by comparison slightly built, is yet over six feet, and, in the hardest of condition, well above twelve stone in weight. Vardon is also between twelve and thirteen stone. Mr. Tait, another six-footer, was a good Rugby football forward, and a glance at his photographs shows that he had amply the physique of one. In fine, the average of physical development of these five long drivers would almost equal that of a brigade of Guardsmen.

It would not be hard to supply the complemental argument. People who can play the short game as well as Mr. J. L. Low and Willie Aveston are not likely to have much to learn in the art of timing the impact of the club. Yet both are relatively short drivers, and both of relatively inconsiderable physique. Rowland Jones's style seems ideal for timing or anything else. There are many bigger men than he though, and many too (of his class of course), who can make him play the odd. Take as a contrast to this the frequent instance of a long handicap man, rather a novice at the game, but well endowed with brawn, the lumbering forward sway of whose big body seems of far more practical effect in moving the ball, when he gets fairly hold of it, than the quickest snap of a light-weight's wrists. And, indeed, this is what one might expect. If the weight of the club and of the ball, like that of the elephant in the Cambridge problem, could be "neglected," as also air resistance, then mere velocity might be the sole determining factor. But as things are, it would seem that for a long drive great momentum, not great velocity alone, of the club-head is required. And momentum has much to do with mass. That was a very discerning, if quite inexcusable, remark of a candid professional to a rather spare patron trying to drive, "Ah, you're too much of a grey-'ound for golf!" On this view, Mr. Hutchinson's consumptive might be like those famous patients of Bodington in the 'fifties, a neglected early example of the benefits of open-air treatment. At all events, there seems much common sense in the summing up of the matter given by the old sporting maxim: "A good big 'un will beat a good little 'un." Perhaps the next Medical Golf Tournament may furnish instances in point.

